# Improved fed-batch processes with *Wickerhamomyces anomalus* WC 1501 for the production of d-arabitol from pure glycerol

**DOI:** 10.1186/s12934-022-01898-y

**Published:** 2022-09-05

**Authors:** Stefano Raimondi, Giorgia Foca, Alessandro Ulrici, Lorenza Destro, Alan Leonardi, Raissa Buzzi, Francesco Candeliere, Maddalena Rossi, Alberto Amaretti

**Affiliations:** 1 Department of Life Sciences, University of Modena and Reggio Emilia, 41125 Modena, Italy; 2 Department of Chemical and Geological Sciences, University of Modena and Reggio Emilia, 41125 Modena, Italy; 3 Department of Chemical, Pharmaceutical and Agricultural Sciences, University of Ferrara, 44121 Ferrara, Italy; 4 Biogest-Siteia, University of Modena and Reggio Emilia, 42124 Reggio Emilia, Italy

**Keywords:** Arabitol, Glycerol, *Wickeramomyces anomalus*, Biorefinery, Central composite design

## Abstract

**Background:**

d-Arabitol, a five-carbon sugar alcohol, represents a main target of microbial biorefineries aiming to valorize cheap substrates. The yeast *Wickerhamomyces anomalus* WC 1501 is known to produce arabitol in a glycerol-based nitrogen-limited medium and preliminary fed-batch processes with this yeast were reported to yield 18.0 g/L arabitol.

**Results:**

Fed-batch fermentations with *W. anomalus* WC 1501 were optimized using central composite design (CCD). Dissolved oxygen had not a significant effect, while optimum values were found for glycerol concentration (114.5 g/L), pH (5.9), and temperature (32.5 °C), yielding 29 g/L d-arabitol in 160 h, a conversion yield of 0.25 g of arabitol per g of consumed glycerol, and a volumetric productivity of 0.18 g/L/h. CCD optimal conditions were the basis for further improvement, consisting in increasing the cellular density (3✕), applying a constant feeding of glycerol, and increasing temperature during production. The best performing fed-batch fermentations achieved 265 g/L d-arabitol after 325 h, a conversion yield of 0.74 g/g, and a volumetric productivity of 0.82 g/L/h.

**Conclusion:**

*W. anomalus* WC 1501 confirmed as an excellent producer of d-arabitol, exhibiting a remarkable capability of transforming pure glycerol. The study reports among the highest values ever reported for microbial transformation of glycerol into d-arabitol, in terms of arabitol titer, conversion yield, and productivity.

**Graphical Abstract:**

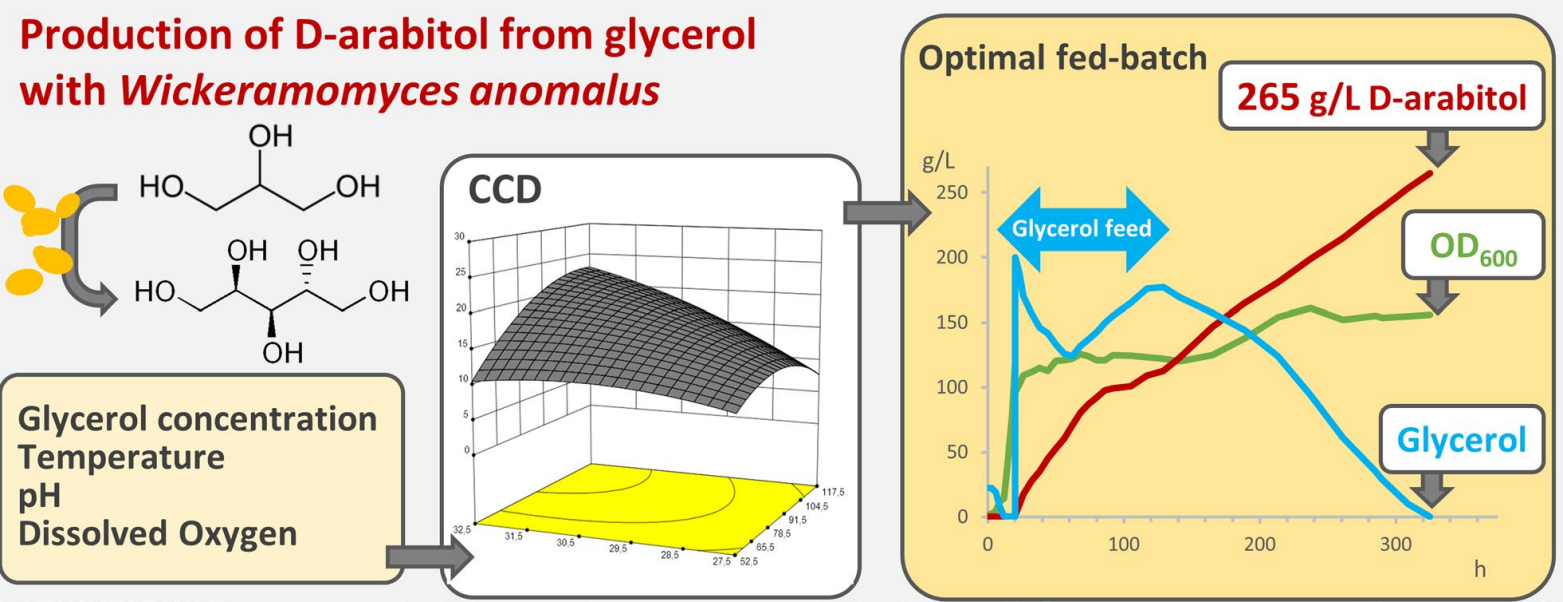

**Supplementary Information:**

The online version contains supplementary material available at 10.1186/s12934-022-01898-y.

## Background

Glycerol is an unavoidable by-product of the biofuel industry and is becoming an attractive feedstock for biorefinery due to its abundance, low price, and high degree of reduction [1]. Biological approaches that convert glycerol into more valuable products have been receiving increasing attention and several microbial processes, utilizing wild type or engineered strains, have been described in the last decade. Promising fed-batch processes have already been shown to produce high yields of the most important targets of glycerol valorization, such as ethanol, diols (1,3-propanediol and 2,3-butanediol), organic acids (D-lactate and hydroxypropionate), and triacylglycerols [2–7]. Considerable efforts have also been devoted to the development of a fermentative process for the transformation of glycerol to compounds such as amino acids, polyhydroxyalkanoates, and polyunsaturated fatty acids [1, 8]. The strain *Wickerhamomyces anomalus* WC 1501 produces polyalcohol arabitol in a glycerol-based nitrogen-limited medium and has thus attracted attention as a strain for a potential transformation of glycerol into a value-added chemical [9].

Sugar alcohols such as xylitol and arabitol have been ranked among the top 12 value added chemicals derivable from biomasses [10, 11]. Arabitol, in particular, is a low-calories anti-cariogenic sweetener and can also serve as substrate or building block in chemical synthesis of several products [10]. The ability of some fungi to produce arabitol from glucose is well established and efficient processes have been proposed [12–14]. Much less information is available on microbial processes yielding arabitol from glycerol [15–18], even though highly performing processes have been recently proposed with *Debaryomyces prosopidis* and *Yarrowia lipolytica* [19, 20]. *W. anomalus* WC 1501 emerged as an arabitol producer from a screening of non-conventional yeasts [9] and was the subject of a preliminary study assessing that arabitol production was not growth-associated and occurred during the stationary phase in presence of an excess of glycerol [21]. Therein, the strain was utilized in a preliminary fed-batch process, where growth and production phases were separated. Growth was carried out in a balanced medium, then production was triggered by a pulse of concentrated glycerol (140 g/L) that was progressively transformed into arabitol, to achieve a final yield of 18.0 g/L [21]. Such process allows to carry out distinct growth and production phases that could be optimized separately. In the present study we aimed to optimize the production phase without affecting the growth performance, focusing on the optimization of production conditions utilizing a central composite design (CCD) to model arabitol production as a function of relevant parameters such as temperature, pH, dissolved oxygen tension (DOT), and glycerol concentration.

## Results and discussion

### Optimization of the production phase with CCD models

A CCD approach was utilized to improve arabitol production and glycerol conversion yield (i.e. the ratio between the mass of arabitol produced and glycerol consumed) in fed-batch processes of *W. anomalus* WC 1501. The experimental design involved the study of 4 process parameters (i.e. glycerol concentration, temperature, pH, and DOT) that could each assume 5 levels (Table 1). The fermentations were started batchwise in a balanced medium (MY) where the yeast was enabled to grow, then different combinations of the process parameters were applied to the production phase, as summarized in Table 2. Within the ranges of the input variables, the different fermentation runs significantly differed in both arabitol production and glycerol conversion yield (Table 2). Both the responses to the independent variables were modeled by a quadratic function harboring a linear and a quadratic coefficient for each variable and an interaction coefficient for each pair of variables. ANOVA results of the fitted quadratic models are shown in Table 3, that reports only the terms retained as significant based on p-values (P < 0.05). For both models no response transformation was necessary and the normality of residuals was verified.

**Table 1:** Coded and real values for each variable of the central composite design

		− α	− 1	0	+ 1	+ α
A	Glycerol, g/L	20.0	52.5	85.0	117.5	150
B	Temperature, °C	25.0	27.5	30.0	32.5	35.0
C	pH	2.5	3.63	4.75	5.87	7.0
D	DOT, %	10	20	30	40	50

**Table 2:** CCD matrix and corresponding response values

Run order	Point type	A	B	C	D	Arabitol (g/L)	Yield (g/g)
1	Factorial	+ 1	− 1	− 1	+ 1	3.4	0.070
2	Axial	0	+ α	0	0	20.1	0.253
3	Axial	− α	0	0	0	1.8	0.077
4	Center	0	0	0	0	16.2	0.220
5	Axial	0	0	0	− α	15.6	0.196
6	Factorial	+ 1	+ 1	− 1	+ 1	18.9	0.204
7	Factorial	− 1	− 1	+ 1	+ 1	6.6	0.134
8	Axial	0	-α	0	0	4.5	0.079
9	Center	0	0	0	0	17.4	0.212
10	Factorial	− 1	+ 1	− 1	− 1	7.3	0.147
11	Axial	0	0	− α	0	19.2	0.250
12	Factorial	− 1	+ 1	+ 1	− 1	12.4	0.244
13	Center	0	0	0	0	19.0	0.236
14	Factorial	+ 1	+ 1	+ 1	− 1	24.0	0.293
15	Factorial	+ 1	− 1	+ 1	− 1	10.2	0.177
16	Factorial	+ 1	− 1	+ 1	+ 1	14.7	0.251
17	Axial	0	0	+ α	0	20.4	0.246
18	Factorial	− 1	− 1	− 1	− 1	6.4	0.137
19	Factorial	− 1	+ 1	+ 1	+ 1	11.7	0.205
20	Center	0	0	0	0	19.0	0.228
21	Factorial	+ 1	+ 1	− 1	− 1	21.0	0.199
22	Factorial	− 1	− 1	− 1	+ 1	5.9	0.121
23	Axial	+ α	0	0	0	8.6	0.189
24	Factorial	+ 1	− 1	− 1	− 1	1.9	0.068
25	Center	0	0	0	0	16.0	0.221
26	Factorial	− 1	− 1	+ 1	− 1	12.0	0.253
27	Axial	0	0	0	+ α	20.0	0.230
28	Factorial	+ 1	− 1	+ 1	+ 1	28.0	0.291
29	Factorial	− 1	+ 1	− 1	+ 1	6.6	0.140
30	Center	0	0	0	0	18.3	0.240

Response values are those registered at the time point when glycerol was depleted or, at the latest, after 140 h of fermentation.

**Table 3:** ANOVA results for the quadratic models describing arabitol concentration and glycerol conversion yield as function of glycerol concentration (A), temperature (B), pH (C), and DOT (D) together with model performance parameters

Response	Source	Sum of Squares	Degrees of freedom	Mean Square	*F* value	p-value Prob > *F*
Arabitol	Model	1272.84	6	212.14	31.51	< 0.0001
	A	185.93	1	185.93	27.61	< 0.0001
	B	416.67	1	416.67	61.88	< 0.0001
	C	106.68	1	106.68	15.84	0.0006
	AB	186.32	1	186.32	27.67	< 0.0001
	A^2^	331.54	1	331.54	49.24	< 0.0001
	B^2^	76.42	1	76.42	11.35	0.0027
	Residual	154.86	23	6.73		
	Lack of fit	145.90	18	8.11	4.53	0.0512
	Pure error	8.95	5	1.79		
	R-squared					0.89
	Adj R-squared					0.86
	Pred R-squared					0.78
	Adeq precision					21.6
Conversion yield	Model	0.090	6	0.015	10.83	< 0.0001
	A	6.534·10^− 3^	1	6.534·10^− 3^	4.72	0.0404
	B	0.031	1	0.031	22.25	< 0.0001
	C	0.024	1	0.024	17.10	0.0004
	AB	6.806·10^− 3^	1	6.806·10^− 3^	4.91	0.0368
	A^2^	0.017	1	0.017	12.23	0.0019
	B^2^	7.425·10^− 3^	1	7.425·10^− 3^	5.36	0.0299
	Residual	0.032	23	1.385·10^− 3^		
	Lack of fit	0.031	18	1.739·10^− 3^	15.62	0.0032
	Pure error	5.568·10^− 4^	5	1.114·10^− 4^		
	R-squared					0.74
	Adj R-squared					0.67
	Pred R-squared					0.52
	Adeq precision					11.1

Only the terms with significant effect (P < 0.05) and those that have significant interactions were retained in the model and reported herein

The model describing arabitol concentration was highly significant, with an F value of 31.51. The performance parameters R-squared, Adj R-squared, and Pred R-squared were all adequate (0.89, 0.86, and 0.78, respectively) and Adeq precision (21.6) indicated that signal to noise ratio of the model is adequate to explore the design space (Table 3). All the linear factors except DOT had significant effect and glycerol-temperature interaction and the quadratic effects of glycerol concentration and temperature were significant as well.

The model describing the conversion yield was also significant, even though all performance parameters were lower than those of the arabitol concentration model. The lack of fit was significant, i.e., the model error was not given only by the random variation but by the fact that the quadratic model did not adequately fit the data, although it was better than the linear one. Any transformation of the response, the search for outliers or the reduction of the model terms did not lead to the solution of this flaw, therefore the model reported was the best obtainable. All the linear factors except DOT, glycerol-temperature interaction and the quadratic effects of glycerol concentration and temperature had significant effect and were maintained also in this model.

The quadratic model equations for arabitol concentration and for the conversion yield are reported Table 4, where Eqs. (1) and (3) require the factors expressed in the original units, while Eqs. (2) and (4) require coded values and are suitable to compare the relative impact of each factor. The first-order term exerting the strongest effect on both arabitol production and glycerol conversion yield was temperature, although also an increase of initial glycerol concentration and of the pH have a positive influence. Among the second-order terms, the quadratic term of glycerol concentration and the glycerol concentration × temperature interaction were very influential on arabitol titer. Such indication on a positive major effect of glycerol is in accord with previous studies consistently indicating that high concentrations of glycerol (or other carbon source) were positively associated with arabitol production [14–16, 18], even though a search of the optimum concentration has not been carried out so far with CCD approach. The not significant effect of DOT is also coherent with literature describing low oxygen demands during arabitol production with other yeasts, except for very low dissolved oxygen concentrations or anaerobic conditions inhibiting the polyol yield [15, 19].

**Table 4:** Quadratic model equations describing arabitol production (g/L) and arabitol/glycerol conversion yield (g/g) as function of the significant factors and their interactions, expressed in their original units (Eqs. 2 and 4) or in coded values (Eqs. 3 and 5)

$${\text{Arabitol }} = -200.85 - 0.62\cdot{\text{Glycerol }} + {{ 13}}.{{83}}\cdot{{T }} + {{ 1}}.{{87}}\cdot{\text{pH }} + {{ }}0.0{{4}}\cdot{{Glycerol}}\cdot{\text{T }}{-}{{ 3}}.{{23}}\cdot{{1}}{0^{ - {{3}}}}\cdot{\text{Glycero}}{{\text{l}}^{{2}}}{-}{\text{ }}0.{{26}}\cdot{{\text{T}}^{{2}}}$$	(1)
$${\text{Arabitol }} = + {{17}}.{{61 }} + {{2}}.{{78}}\cdot{\text{A }} + {{ 4}}.{{17}}\cdot{\text{B }} + {{ 2}}.{{11}}\cdot{\text{C }} + {{ 3}}.{{41}}\cdot{\text{AB }}{-}{{ 3}}.{{41}}\cdot{{\text{A}}^{{2}}}{-}{{ 1}}.{{64}}\cdot{{\text{B}}^{{2}}}$$	(2)
$${\text{Yield }} = {\text{ }}{-}{{ 2}}.{{23 }}{-}{{ 3}}.{{18}}\cdot{{1}}{0^{ - {{3}}}}\cdot{\text{Glycerol }} + {\text{ }}0.{{15}}\cdot{\text{T }} + {\text{ }}0.0{{3}}\cdot{\text{pH }} + {{ 2}}.{{54}}\cdot{\text{Glycerol}}\cdot{\text{T}}{-}{{ 2}}.{{31}}\cdot{{1}}{0^{ - {{5}}}}\cdot{\text{Glycero}}{{\text{l}}^{{2}}}{-}{{ 2}}.{{58}}\cdot{{1}}{0^{ - {{3}}}}\cdot{{\text{T}}^{{2}}}$$	(3)
$${\text{Yield }} = {\text{ }} + {\text{ }}0.{{23 }} + {\text{ }}0.0{{17}}\cdot{\text{A }} + {\text{ }}0.0{{36}}\cdot{\text{B }} + {{ }}0.0{{31}}\cdot{\text{C }} + {\text{ }}0.0{{21}}\cdot{\text{AB }}{-}{\text{ }}0.0{{24}}\cdot{{\text{A}}^{{2}}}{-}{\text{ }}0.0{{16}}\cdot{{\text{B}}^{{2}}}$$	(4)

**Fig. 1:**
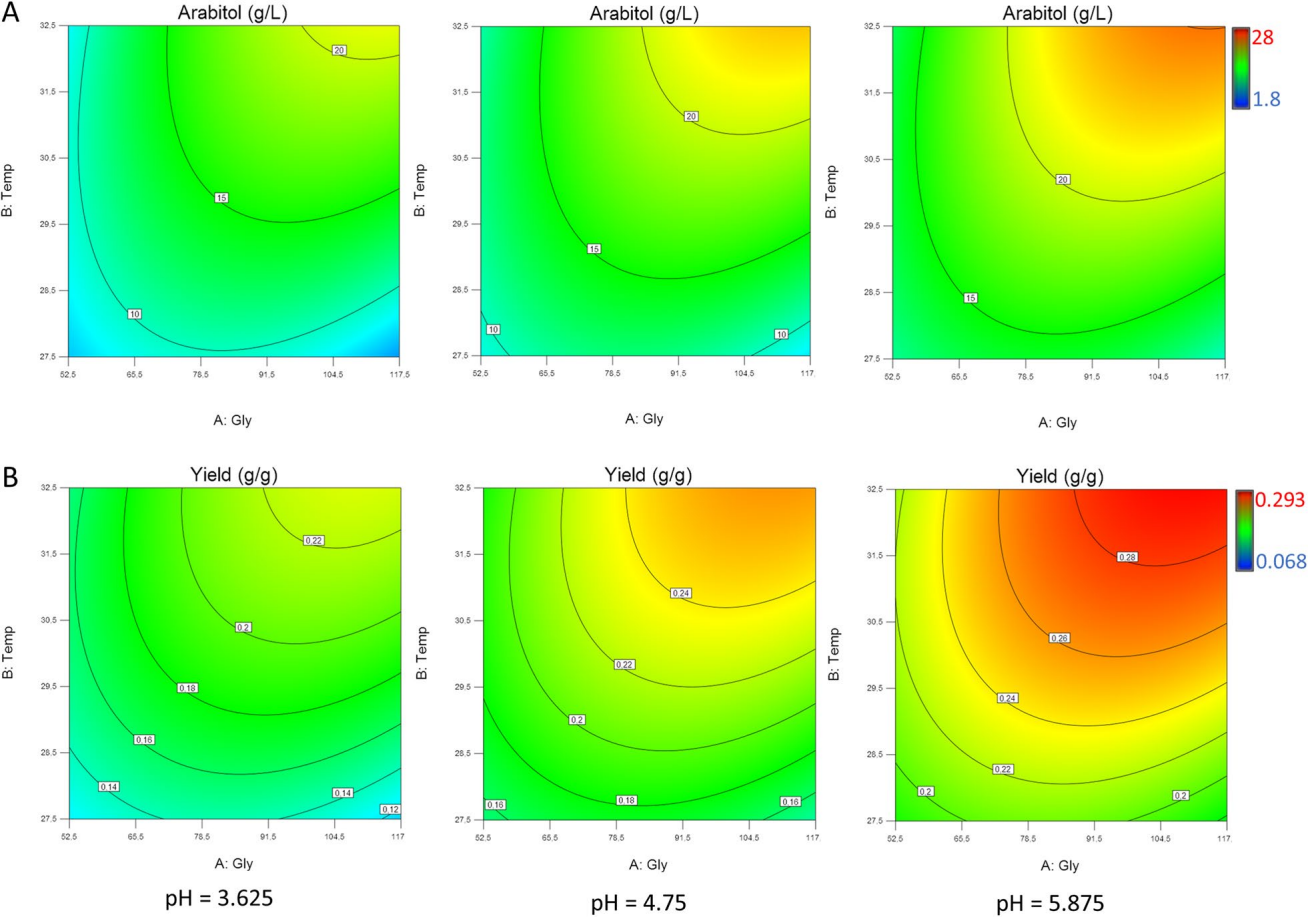
Response surface plot for arabitol concentration (**a**) and conversion yield (**b**) on varying glycerol concentration, temperature, and pH (DOT = 30%)

The response surface of arabitol production and conversion yield as a function of temperature, glycerol, and pH are reported in Fig. 1. Numerical optimization was carried out in search of set of factors that would lead to the best compromise solution, simultaneously maximizing both arabitol and yield responses. In computing the desirability function the responses were given importance values of 5 and 2, respectively, based on their model reliability. Exploration of the design space yielded the best optimum solution for following factors setting: glycerol concentration = 114.5 g/L, temperature = 32.5 °C, pH = 5.875 and dissolved oxygen tension = 31.2%. In these conditions, predicted responses were: arabitol concentration = 25.06 g/L and conversion yield = 0.29 g/g. Triplicate fed-batch fermentations were carried out to validate the optimal factors.

**Fig. 2:**
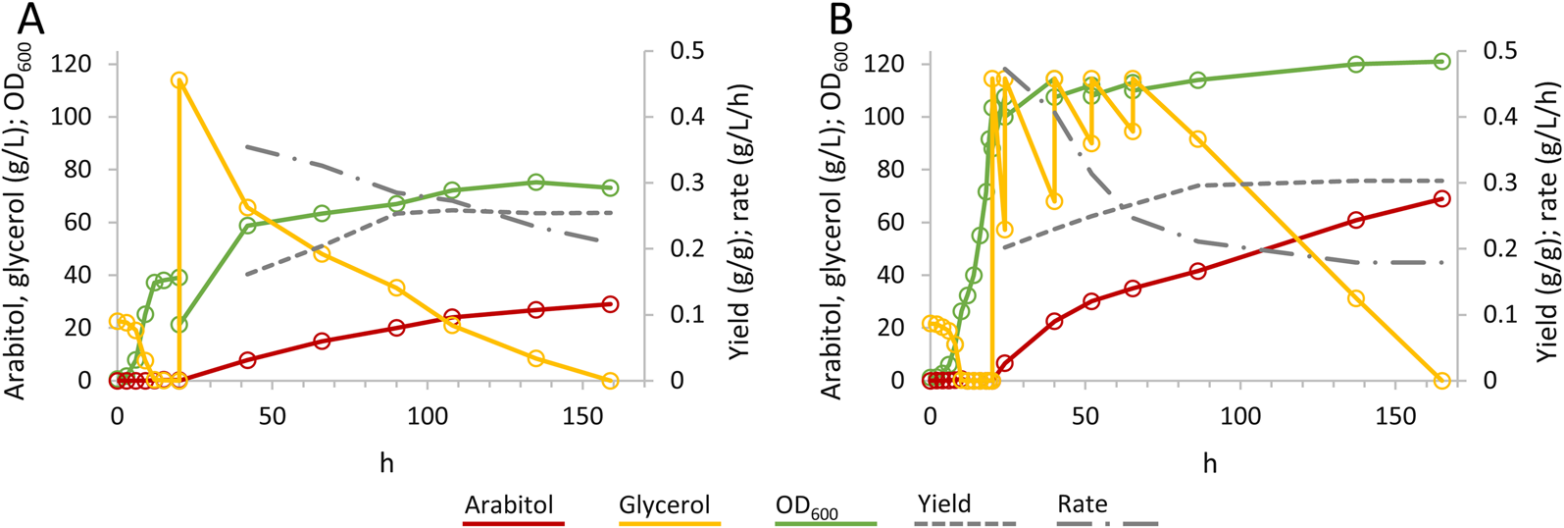
Time-course of fed batch fermentations, carried out under the optimal conditions (glycerol concentration = 114.5 g/L, temperature = 32.5 °C, pH = 5.875 and DOT = 31.2%).** A** Fed batch process utilized for model validation.** B** Fed batch process with extended growth phase (3✕) and with application of repeated glycerol pulses. The experiments were carried out in triplicate. Representative time-courses are reported herein

The results were elaborated using the t-test (P = 95%, n = 3) to compare the experimental mean obtained from the replicates with the expected value of the model. The experimental mean for both arabitol concentration and conversion yield obtained after 140 h of fermentation lay within the prediction intervals, i.e., did not significantly differ from the predicted values, confirming the accuracy of the calculated models (Table 5). Fermentation runs were then allowed to deplete glycerol, that was exhausted at 160 h, with a final production of 29 g/L, a conversion yield that reached a plateau of 0.25 g/g, a production rate of 0.21 g/L/h, and an overall volumetric productivity of 0.18 g/L/h (Fig. 2A).

**Table 5:** Results of the validation experiment compared to the predicted value

Response	Experimental data mean	Experimental standard deviation	Lower prediction limit (P = 95%)	Predicted value	Higher prediction limit (P = 95%)
Arabitol (g/L)	24.40	2.59	21.12	25.06	29.00
Yield (g/g)	0.26	0.04	0.24	0.29	0.34

The supernatant of the fed-batch cultures was lyophilized overnight. The resulting orange solid was confirmed to be > 95% arabitol by ^1^HNMR (Additional file 1: Figure S1) and identified as d-arabitol by polarimetric analysis.

### Further improvements of the fed batch processes

In order to increase the amount of cells and thus the production rate, the growth phase was tried in three-fold concentrated MY medium, but the oxygen demand resulted excessive. Therefore, concentrated MY medium was fed at limited rate, equally providing the culture with a triple amount of all the MY medium components. At the end of the enhanced growth phase, the culture contained 5.5 ✕10^9^ cells/mL, 33.6 g/L biomass and < 0.5 g/L arabitol, and depleted both glycerol and ammonium. The production phase was then induced in grown cultures, providing concentrated glycerol in different modes. All the fermentations were carried out in triplicate and a representative runs are described below. For each feeding mode, the final arabitol titer, the yield, and the productivity differed by less than 10%.

First, the optimal production conditions identified by CCD were applied to the grown cultures with extended growth phase. Between 24 and 72 h of fermentation, the cultures received 5 pulses of concentrated glycerol to bring glycerol concentration to the optimal value of 114.5 g/L (Fig. 2B). After 165 h, *W. anomalus WC 1501* exhausted all the fed glycerol, with a production of 69 g/L d-arabitol. In total, 89 g of glycerol were fed and transformed into 22.5 g of arabitol. Throughout the pulses, the conversion yield progressively increased, reaching a plateau of 0.30 g/g. On the other hand, the production rate progressively decreased from 0.47 g/L/h in the first hours, settling on a value of 0.18 g/L/h. The volumetric productivity throughout the whole process was 0.41 g/L/h.

**Fig. 3:**
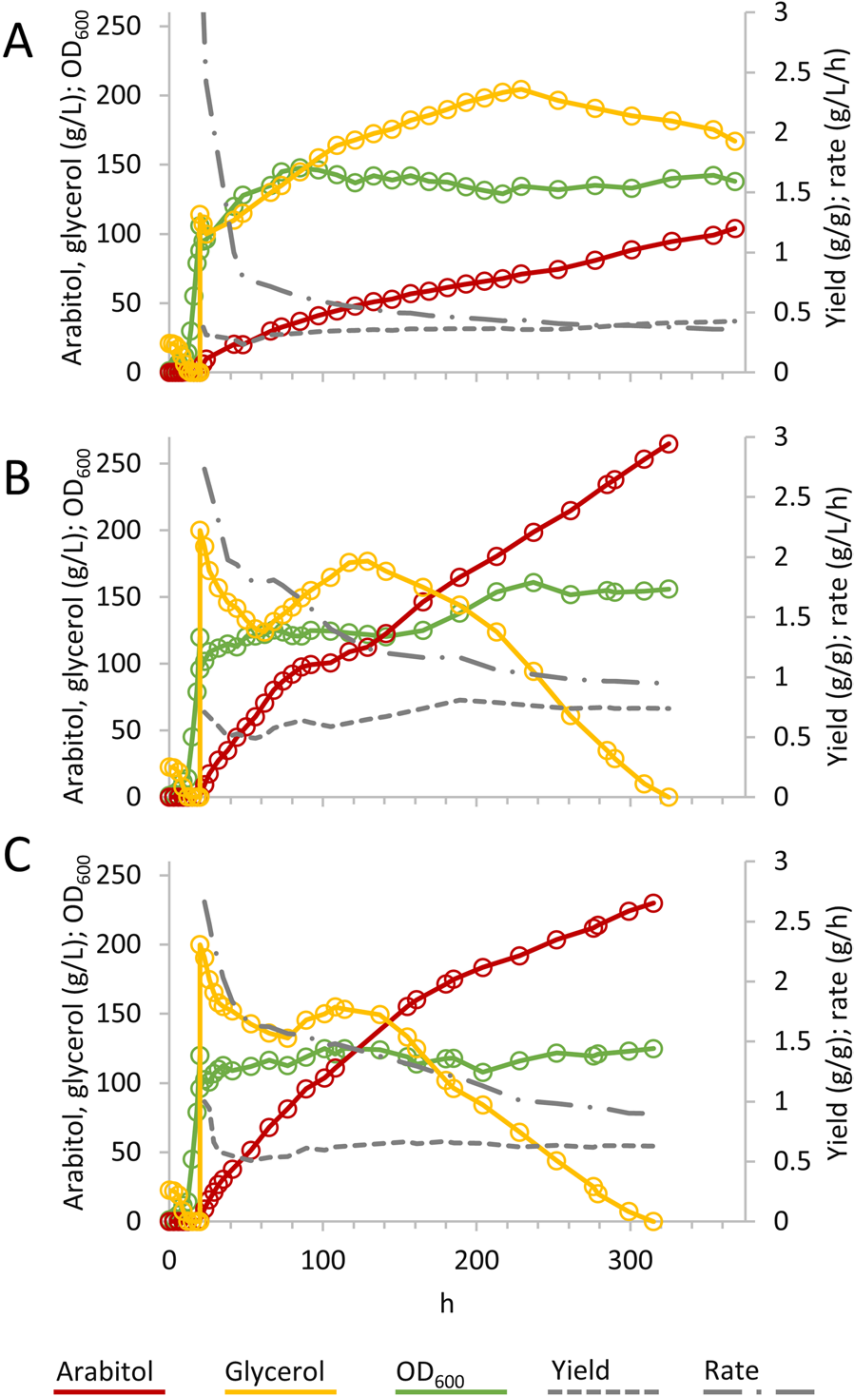
Time-course of improved fed batch fermentations. In all the fermentations the growth phase was extended (3✕). In the production a total of 200 g of glycerol was supplied through an initial pulse and followed by continuous feeding at 1.75 g/L/h. **A** Pulse of 114.5 g/L glycerol, 32.5 °C. **B** Pulse of 200 g/L glycerol, 36 °C. **C** Pulse of 200 g/L glycerol, 38 °C. Optimal pH and DOT values were maintained. The experiments were carried out in triplicate. Representative time-courses are reported herein

After the pulse of concentrated glycerol, continuous feeding, instead of pulsed, was applied to cultures with extended growth phase, providing the culture with 200 g of glycerol in total (Fig. 3). In a first attempt (Fig. 3A), a pulse of 114.5 g/L glycerol was applied, followed by a continuous feeding of 1.75 g/L/h glycerol until a total of 200 g were given to the culture after 210 h. The pH and temperature were set at the optimal values. Glycerol was initially rapidly consumed, then progressively accumulated up to 204 g/L and was eventually consumed when feeding was interrupted after 210 h of fermentation. Arabitol concentration was 72 g/L at 210 h and increased to 104 g/L at 360 h, when the fermentation was interrupted. At the end of the fermentation 175 g/L glycerol remained unconsumed. The conversion yield was 0.43 g/g. The production rate was remarkably high in the first hours of feeding (approx. 3 g/L/h), then decreased abruptly and settled on a value of 0.36 g/L/h. Throughout the process, a volumetric productivity of 0.29 g/L/h was achieved. The turbidity of the culture increased during the first hours of the production phase but was not accompanied by any increase in cell numbers.

Further experiments indicated that increasing the temperature had the effect of increasing glycerol consumption rate in the initial hours of the production phase and the culture could cope with higher glycerol pulses (data not shown) without getting flooded by the carbon source. In a process where the production phase was carried out at 36 °C (Fig. 3B), glycerol was given with a pulse of 200 g/L and continuously fed at the rate of 1.75 g/L/h until 129 h, when the 200 g of glycerol were fed. At this time point, the culture had already produced 112 g/L arabitol and glycerol, that had initially decreased to 123 g/L, had risen to 177 g/L. The residual glycerol continued to be consumed and got exhausted after 325 h of fermentation, when arabitol reached the concentration of 265 g/L. During the production phase, the conversion yield and rate tended to 0.74 g/g and 0.95 g/L/h, respectively. The process performed a volumetric productivity 0.82 g/L/h.

In a similar process where the production phase was carried out at 38 °C (Fig. 3C), the culture presented the same trend, but with a lower rise of glycerol (155 g/L after 129 h). Such process presented a lower efficiency, yielding 230 g/L arabitol after 315 h, with a conversion yield of 0.64 g/g, a rate of 0.90 g/L/h, and a volumetric productivity of 0.73 g/L/h.

These best process among the ones herein described achieved the highest values of arabitol titer and conversion yield ever reported for a yeast in a glycerol based medium and a remarkably high productivity as well (Table 6) [16, 17, 19, 20], indicating *W. anomalus* WC1501 as a good candidate to be challenged in a process with crude glycerol. These results indicated that maintaining high glycerol concentration was pivotal for triggering arabitol production by *W. anomalus* WC 1501, confirming the evidence of previous studies [16, 17, 19, 20]. Another feature that this study shares with all the previous ones reporting high arabitol production is the fact that the high concentration of the carbon source was accompanied by limiting concentrations of the nitrogen source, in conditions of high C/N ratio [14–21]. Similar conclusions were obtained recent studies with the *Debaryomyces prosopidis* and *Yarrowia lipolytica*, reporting very performing fermentative processes [19, 20]. Fed-batch culturing fulfilled the purpose of maintaining high glycerol concentration, as already described with a *Y. lipolytica* strain that achieved remarkably high values of arabitol concentration (118.5 g/L), yield (0.49 g/g), and productivity (1.10 g/L/h) [20]. However, unlike in *W. anomalus* WC 1501, arabitol production by *Y. lipolitica* was coupled with cell growth and the effect of glycerol concentration was interpreted as an adaptation to osmotic stress.

**Table 6:** Comparison of yeast processes transforming glycerol into arabitol. Mode: SF, shake flasks; B, batch in bioreactor; FB fed-batch

Strain	Glycerol	Cultivation mode	Arabitol g/L	Yield g/g	Volumetric productivity g/L/h	References
*Debaryomyces hansenii* NRRL Y-7483	Pure	B	40	0.55	0.33	[16]
*Candida quercitrusa* NBRC1022	Crude	B	85	0.41	0.35	[17]
*Debaryomyces prosopidis* FMCC Y69	Crude	SF	57	0.48	0.11	[19]
*Yarrowia lipolytica* ARA9	Crude	FB	119	0.49	1.10	[20]
*Wickerhamomyces anomalus* WC 1501	Pure	FB	265	0.74	0.82	This study

In the processes with *W. anomalus* WC 1501 described in the present and previous studies, arabitol generation mainly occurred during the stationary phase under conditions of nitrogen limitation, with constant number of biocatalytic cells linearly converting an excess of glycerol into arabitol. Consistently, the rate of production seemed mainly related to the concentration of biocatalytic cells obtained during the growth phase, as indicated by enhanced-growth fed-batch processes, with temperature exerting a positive effect, within certain limits, on the transformation kinetics. Some features of *W. anomalus* can be hypothesized to explain the high arabitol concentration, glycerol conversion yield, and productivity. In fact, *W. anomalus* possesses efficient glycerol transport system, able to fuel fast metabolism of this carbon source, and cyanide-resistant respiration (CRR) machinery that could get rid of the reduced cofactors generated along with arabitol, yielding a low proton motive force under conditions characterized by growth limitation and thus by low ATP demand [22]. The impact of high osmolarity on growth performance and productivity has not yet been specifically studied in *W. anomalus* WC1501. Thus, it remains to be clarified whether the osmotic stress itself was involved in arabitol production, as it had in *Y. lipolytica* but had not in *D. hansenii* [15, 20], and could be a further target of investigation and process improvement.

The metabolic relationship between arabitol production and the accumulation of storage triacylglycerols is another subject deserving deeper investigation, as they take place under similar conditions. In fact, the high C/N ratio required for efficient arabitol production is the same that induce the accumulation of storage lipids in oleaginous yeasts, including *Y. lipolytica* [23], but it remains to be clarified whether arabitol generation is somehow functional to lipids biosynthesis. *W. anomalus* WC 1501 itself presented some ability to accumulate triacylglycerols within lipids bodies that reached up to the 23% of biomass dry weight [21]. Nonetheless, lipid yield was not significantly affected throughout the CCD (data not shown).

## Conclusion

The present study reports among the highest values ever reported for microbial transformation of glycerol into d-arabitol, in terms of arabitol titer, conversion yield, and productivity. The strain *W. anomalus* WC 1501 confirmed as an excellent producer of d-arabitol, exhibiting a remarkable capability of transforming pure glycerol. The strain represents a good candidate to be challenged with raw glycerol from biodiesel industry and deserves deeper investigation in the perspective of finding application in such microbial biorefinery.

## Methods

### Strain, media and chemicals

*W. anomalus* WC 1501 was obtained from our laboratory collection. The strain was routinely cultured at 30 °C in shake-flasks of YPD broth (BD Difco, Sparks, MD, USA) and maintained at 4 °C in agar slants of YPD. MY medium, containing 20 g/L pure glycerol, 3 g/L yeast extract (BD Difco, Sparks, MD, USA), 2 g/L (NH_4_)_2_SO_4_, 3 g/L KH_2_PO_4_, 1 g/L K_2_HPO_4_, and 1 g/L MgSO_4_ · 7H_2_O, was utilized to prepare the seed culture for bioreactors. All chemicals were purchased from Sigma-Aldrich (Steinheim, Germany), unless otherwise stated.

### Bioreactor operation and central composite design fed-batch experiments

Fed-batch fermentations were carried out in laboratory-scale autoclavable bioreactors (500 mL Mini Bio, Applikon Biotechnology, Delft, the Netherlands). Processes were started batch-wise in 400 mL MY medium, inoculated 5% (v/v) with a seed culture grown at 30 °C for 24 h. The culture was maintained at 30 °C and aerated with 0.5 v/v/min filter-sterilized air. Automatic addition of 4 M NaOH prevented the pH was from decreasing below 5.0. Cascade-controlled stirring from 1000 to 1700 rpm was used to maintain the dissolved oxygen tension (DOT) at 20%. A defoaming mixture (1:1, v/v) of Xiameter 1520 (Dow Corning, Midland, MI, USA) and polypropylene-glycol was automatically added to prevent foaming. After 12 h of batch growth, the production phase was started under the conditions of glycerol, pH, temperature and DOT planned in the experimental design for the specific fermentation run (Tables 1 and 2). The volume was adjusted to 250 mL and was supplemented with 150 mL of glycerol solution, in order to supply the culture with the desired concentration. The pH was adjusted and its set point was changed accordingly. The culture was periodically sampled, in order to monitor biomass, glycerol, and arabitol concentration.

The conversion yield was expressed as the grams of arabitol per gram of glycerol consumed. Instantaneous production rate was calculated considering, for each time interval, the volume change due to the feeding and the amount of arabitol withdrawn with the sampling. Volumetric productivity was calculated as the final arabitol titer, divided by the time.

### Design of experiments

The experimental design was set up with Design Expert software (Version 10 Stat-Ease Inc., USA), with the aim to optimize the production phase of fed batch processes of *W. anomalus* WC 1501. Glycerol concentration at the beginning of the production phase, temperature, pH, and DOT were the four factors taken into account in a second order symmetrical design. Two responses were considered: arabitol concentration and the conversion yield, both evaluated in correspondence of glycerol exhaustion or after 140 h in case the carbons source was not depleted. A Central Composite Design (CCD) was utilized, with 5 levels for each factor coded as − α, − 1, 0, + 1, + α (Table 1). The design was composed of 30 experimental runs, conducted in random order, consisting in 16 factorial and 8 axial combinations and 6 repetitions of the central point (Table 2).

The responses to the independent variables were modeled by a quadratic function according to the general equation:

5 $$Y = {\beta _0} + \sum _{i = 1}^k{\beta _i}{X_i} + \sum _{i = 1}^k{\beta _{ii}}X_i^2 + \sum _{1 \le i \le j}^k{\beta _{ij}}{X_i}{X_j}$$

where *Y* is the measured response variable, *k* is the number of factors, *β*_*0*_ is the constant term, *β*_*i*_, *β*_*ii*_ and *β*_*ij*_ are, respectively, the linear, quadratic and interaction coefficients, *X*_*i*_ and *X*_*j*_ represent the factors [24].

The significance of each model term was verified by means of the analysis of variance (ANOVA) and the variation sources were compared by using the Fisher distribution (F test) with p < 0.05. The computation also yielded the following performance parameters: the coefficient of determination of the model (R-squared), the adjusted coefficient of determination (Adj R-squared, i.e. an estimation of the fit considering the number of parameters in the model relative to the number of points in the design), the coefficient of determination in prediction (Pred R-squared), and the adequate precision value (Adeq precision, i.e. an estimation of whether the signal to noise ratio is adequate to explore the design space, with values greater than 4 being desirable).

To evaluate the validity of the models, the numerical optimization for both arabitol concentration and conversion yield was performed by maximizing the desirability function [25]. The resulting validation experiment was tested in triplicate closely around the predicted optimum, and the experimental means of the values measured for arabitol concentration and conversion yield were compared with the corresponding model prediction intervals [26].

### Improved fed-batch fermentations

Cultures were started batch-wise in 360 mL MY medium, modified with 9 g/L KH_2_PO_4_ and 3 g/L K_2_HPO_4_. After 12 h, the culture was fed 36 mL of a solution containing 400 g/L glycerol, 60 g/L yeast extract, 40 g/L (NH_4_)_2_SO_4_, and 20 g/L MgSO_4_, at the rate of 2.5 mL/h. After 20 h, the volume was adjusted to 250 mL, the pH and temperature were adjusted as appropriate for the specific experiment, and production was started with a pulse of 800 g/L glycerol, in order to achieve the desired concentration. During the production, the culture was continuously fed in order to supply the culture with a total of 250 mL glycerol solution (i.e. 200 g of glycerol). Fed-batch fermentations were carried out in triplicate.

### Chemical analysis

Growth was monitored by measuring turbidity at 600 nm (OD_600_), cell counts, and biomass dry weight (DW). Cells were counted in a Bürker chamber; the DW was determined with a thermo-balance (MB 64 M, VWR, Radnor, PA, USA) (20).

Arabitol and residual glycerol in culture supernatants—clarified by centrifugation (10,000 rpm for 5 min at 4 °C) and filtration at 0.22 μm—were analyzed in HPLC with refractive index detector (1200 System, Agilent Technologies, Waldbronn, Germany). Isocratic elution was carried out at 60 °C with 0.8 mL/min of 5 mM H_2_SO_4_ through an ion exclusion column (Aminex HPX-87 H, Bio-Rad, Hercules, CA, USA) [27]. ^1^ H- spectra were recorded at 298 K on Bruker FT-NMR Advance 400 (400.13 MHz). Chemical shift values are given in ppm relative to TMS and were determined by taking as reference the isotopic impurity signals DMSO-d6 (2.50 ppm). Prior to NMR analysis, the supernatant of the improved fed-batch cultures was lyophilized overnight with a Alpha 1–2 LD Laboratory (Christ, Germany). Polarimetric analysis was carried out using a Polax-2 L polarimeter (Atago, Japan).

## Supplementary Information

**Additional file 1: Figure S1**. 1H NMR (400 MHz, DMSO-d6) of the lyophilized supernatant of W.anomalus WC 1501 fed batch culture. δ 3.27 – 3.49 (m, 5H, 1-5), 3.59 (d, J = 10.9 Hz, 1H, 6 or 7),3.66 (d, J = 6.9 Hz, 1H 6 or 7), 4.12 (d, J = 7.2 Hz, 1H 8), 4.19 (bs, J = 5.8 Hz, 1H, 9 or 10), 4.30 (bs,J = 6.6 Hz, 1H, 9 or 10), 4.43 (bs, J = 6.1, 6.7 Hz, 2H, 11, 12).

## Data Availability

All of the material is owned by the authors and/or no permissions are required.
