# Supplementary material for: Improved fed-batch processes with Wickerhamomyces anomalus WC 1501 for the production of d-arabitol from pure glycerol
Source: Microb Cell Fact. 2022 Sep 5;21:179. doi: 10.1186/s12934-022-01898-y (PMC9442996; doi:10.1186/s12934-022-01898-y)
Supplement: Supplementary file 1 — Additional file 1: Figure S1. 1H NMR (400 MHz, DMSO-d6) of the lyophilized supernatant of W.anomalus WC 1501 fed batch culture. δ 3.27 – 3.49 (m, 5H, 1-5), 3.59 (d, J = 10.9 Hz, 1H, 6 or 7),3.66 (d, J = 6.9 Hz, 1H 6 or 7), 4.12 (d, J = 7.2 Hz, 1H 8), 4.19 (bs, J = 5.8 Hz, 1H, 9 or 10), 4.30 (bs,J = 6.6 Hz, 1H, 9 or 10), 4.43 (bs, J = 6.1, 6.7 Hz, 2H, 11, 12). [file 12934_2022_1898_MOESM1_ESM.pdf]

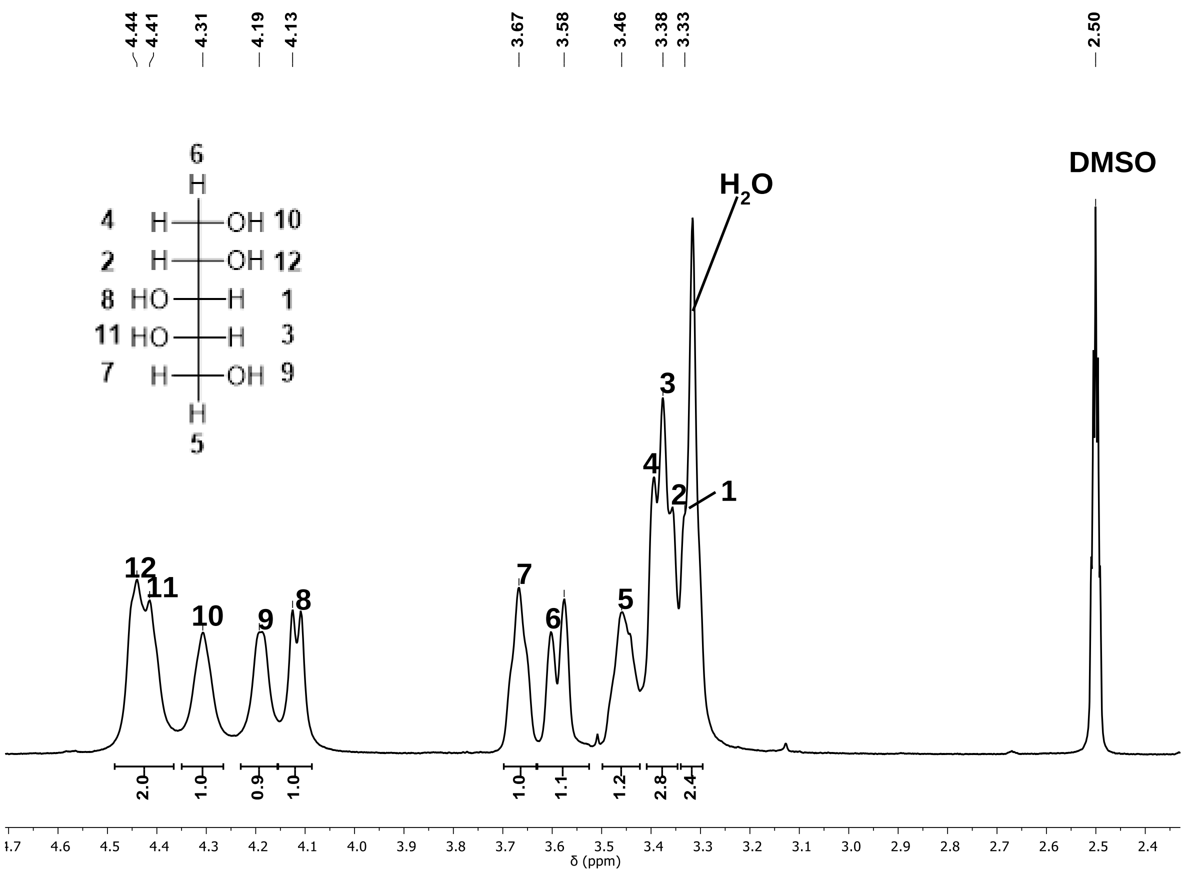

Supplementary Figure S1. ^{1}H NMR (400 MHz, DMSO-d_6) of the lyophilized supernatant of *W. anomalus* WC 1501 fed batch culture. δ 3.27 – 3.49 (m, 5H, **1**-**5**), 3.59 (d, *J* = 10.9 Hz, 1H, **6** or **7**), 3.66 (d, *J* = 6.9 Hz, 1H **6** or **7**), 4.12 (d, *J* = 7.2 Hz, 1H **8**), 4.19 (bs, *J* = 5.8 Hz, 1H, **9** or **10**), 4.30 (bs, *J* = 6.6 Hz, 1H, **9** or **10**), 4.43 (bs, *J* = 6.1, 6.7 Hz, 2H, **11**, **12**).
